# A transient resistance to blood-stage malaria in interferon-γ-deficient mice through impaired production of the host cells preferred by malaria parasites

**DOI:** 10.3389/fmicb.2015.00600

**Published:** 2015-06-17

**Authors:** Hiroko Okada, Kazutomo Suzue, Takashi Imai, Tomoyo Taniguchi, Chikako Shimokawa, Risa Onishi, Jun Hirata, Hajime Hisaeda

**Affiliations:** Department of Parasitology, Graduate School of Medicine, Gunma UniversityMaebashi, Japan

**Keywords:** malaria, IFN-γ, host–parasite relationship, reticulocytes, erythropoiesis

## Abstract

IFN-γ plays both pathological and protective roles during blood-stage malaria. One of its pathological roles is its contribution to anemia by suppressing erythropoiesis. Here, to evaluate the effects of IFN-γ-mediated alterations in erythropoiesis on the course of malaria infection, mice deficient in IFN-γ (GKO) were infected with two strains of the rodent malaria parasite *Plasmodium yoelii*, 17XL (PyL) and 17XNL (PyNL), whose host cell ranges differ. Regardless of genotype, all mice infected with PyL, which can invade any erythrocyte, developed high parasitemia and died quickly. Although PyNL caused a transient non-lethal infection in wild-type (WT) mice, some GKO mice were unable to control the infection and died. However, GKO mice were resistant to the early phase of infection, showing an impaired increase in parasitemia compared with WT mice. This resistance in the GKO mice was associated with having significantly fewer reticulocytes, which are the preferred host cells for PyNL parasites, than the WT mice. Compared with the amount of reticulocytes in GKO mice during the early stages of infection, there was a significant increase in the amount of these cells at later stages, which coincided with the inability of these mice to control the infection. We found that the growth of PyNL parasites correlated with the amount of reticulocytes. Thus, the reduced number of reticulocytes in mice lacking IFN-γ appears to be responsible for the limited parasite growth. Notably, these differences in GKO mice were at least partially reversed when the mice were injected with exogenous IFN-γ. Additionally, an artificial induction of hemolytic anemia and an increase in reticulocytes by phenylhydrazine treatment in GKO mice completely abolished the lower parasitemia and resistance during early phase infection. These results suggest that IFN-γ may contribute to the early growth of PyNL parasites by increasing the amount of reticulocytes, presumably by enhancing erythropoiesis.

## Introduction

Malaria, caused by infection with genus *Plasmodium* protozoan parasites, produces over 200 million infections and approximately 600 thousand deaths every year (WHO, 2014). The major reasons that malaria remains life-threatening are the absence of effective vaccines and the spread of chloroquine-resistant parasites. Unfortunately, developing an effective vaccine for malaria is challenging because malaria infection induces complicated pathological consequences that are formed by both parasite factors and host immune responses. Therefore, to effectively control malaria and successfully develop effective vaccines, it is important to understand the host–parasite interactions in detail.

All malaria symptoms, including fever, anemia, and splenomegaly, appear while the malaria parasites undergo erythrocytic cycles. Although all species of malaria go through these cycles, the host cell specificities of the malaria parasites vary among species. For example, *P. vivax* prefers to infect immature reticulocytes, while *P. malariae* prefers mature red blood cells (White, 1996). Importantly, the host cell preference of rodent malaria parasites influences their virulence. The *P. yoelii* 17XNL parasite infects only reticulocytes and causes a transient infection in wild-type (WT) mice (Jayawardena et al., 1983). In contrast, the *P. yoelii* 17XL strain, a variant derived from *P. yoelii* 17XNL, invades a wide range of erythrocytes, resulting in a lethal infection with high parasitemia (Jayawardena et al., 1983; Otsuki et al., 2009).

Interferon (IFN)-γ is a pro-inflammatory cytokine produced by several cell types, including CD4^+^ T cells, CD8^+^ T cells, γδ T cells, and NK cells (Villegas-Mendez et al., 2012; Inoue et al., 2013). In murine malaria models, several reports demonstrate that this cytokine is indispensable for protection against blood-stage infections. Mice genetically deficient in IFN-γ (GKO) or IFN-γ receptor (γRKO) suffer from prolonged malaria infections or even succumb to otherwise non-lethal malaria infections (Favre et al., 1997; Yoneto et al., 1999). Moreover, dosing WT mice with a neutralizing antibody targeting IFN-γ remarkably attenuated their resistance to malaria parasites (Waki et al., 1992). In contrast, IFN-γ also contributes to pathogenesis during malaria infection. GKO and γRKO mice infected with *P. berghei* ANKA were refractory to the experimental cerebral malaria observed in similarly infected B57BL/6 mice, but were still unable to control the infection with these parasites and died of high parasitemia (Rudin et al., 1997; Villegas-Mendez et al., 2012). Additionally, liver injury in WT mice infected with *P. berghei* NK65 was prevented when the mice were injected with an antibody to IFN-γ (Yoshimoto et al., 1998).

Another important function of IFN-γ is to regulate hematopoiesis during inflammatory processes by affecting both hematopoietic stem cells and their downstream progenitor cells. In general, this pro-inflammatory cytokine and TNF-α are both suppressors of hematopoiesis, and they inhibit the self-renewal of hematopoietic stem cells (Sato et al., 1995). However, IFN-γ does not always act to suppress hematopoiesis; a recent report demonstrated that IFN-γ contributed to the maintenance of hematopoietic stem cells, which supply immune effector cells during chronic bacterial infections in mice (Baldridge et al., 2010). Erythropoiesis is likewise suppressed by IFN-γ. For example, macrophages activated by IFN-γ contribute to a loss of erythrocytes via enhanced hemophagocytic activity (Zoller et al., 2011). Furthermore, IFN-γ inhibits the iron recycling required for effective erythropoiesis (Weiss, 2009). Importantly, IFN-γ directly suppresses erythroid colony formation from hematopoietic stem cells (Raefsky et al., 1985; Broxmeyer et al., 1986), as well as differentiation and proliferation of early erythroid progenitors (Wang et al., 1995). In addition to its immunological roles, IFN-γ may play pivotal roles in the host-parasite relationship during malaria infection by altering erythropoiesis because this process is responsible for the production of host cells for the malaria parasites. However, it remains unknown how IFN-γ-associated alterations in erythropoiesis affect the course of infection.

This study found that GKO mice infected with PyNL showed significantly lower parasitemia in the early phase of infection compared with infected WT mice, even though some GKO mice were unable to completely clear the late stage infection. The partial resistance to early stages of malaria in GKO mice was attributed to the observed absence during this period of the typical increase in reticulocytes, which are the preferred host cells for PyNL. These results suggest that although IFN-γ is generally considered a suppressor of erythropoiesis, it actually enhances erythropoiesis in response to malaria during the early phase of infection.

## Materials and methods

### Experimental animals and parasites

Male and female C57BL/6 mice, used as WT controls, were purchased from Japan SLC (Hamamatsu, Japan) and used for experiments when they were 6–8 weeks old. GKO mice with a C57BL/6 background were kindly provided by Dr. A. Nakane (Hirosaki University). All mice were maintained under specific-pathogen-free conditions. Age- and sex-matched groups were used in each experiment. All mouse experiments were reviewed and approved by the Committee for Ethics on Animal Experiments in the Faculty of Medicine and performed under the control of the Guidelines for Animal Experiments in the Faculty of Medicine, Gunma University, according to Japanese law (no. 105) and notification (no. 6) of the Government of Japan.

*Plasmodium yoelii* 17XNL (PyNL) and 17XL (PyL) were generous gifts from Dr. M. Torii (Ehime University). Recombinant parasites expressing GFP, generated previously (Imai et al., 2013), were also used. These recombinant parasites maintained the virulence of the parental strains; the kinetics of infection with each of the recombinant parasites was exactly the same as with their parental parasites (Imai et al., 2013, data not shown). Blood-stage parasites for experimental infection were obtained from donor mice 2–3 days after injection with frozen stock when these mice showed approximately 5% parasitemia. Experimental mice were given an intraperitoneal infection with 1 × 10^4^ parasitized erythrocytes. Following infection, mouse survival and parasitemia were monitored throughout an observation period, ranging from 9 to 40 days. Parasitemia was determined by counting the percentage of infected erythrocytes in a Giemsa-stained thin blood film under a microscope, using blood from the tail vein of infected mice.

### Flow cytometric analyses

Peripheral blood obtained from tail veins was stained with PE-anti-CD71 and PE-Cy5.5-anti-TER119 (eBioscience, San Diego, CA, USA). Stained cells were analyzed using a FACSCalibur flow cytometer (Becton Dickinson, Mountain View, CA, USA), and data sets were analyzed using FlowJo software (Tree Star, Ashland, OR, USA).

### Injection of IFN-γ

In some experiments, recombinant mouse IFN-γ (PeproTech, Rocky Hill, NJ) was administered intraperitoneally to some of the GKO mice. Ten thousand units of IFN-γ were injected daily from 4 to 13 days after infection with PyNL. The control GKO mice were injected with vehicle (200 μl of saline) alone.

### Induction of reticulocytosis

To induce hemolytic anemia, mice were intraperitoneally injected with 5 mg of phenylhydrazine hydrochloride (PHZ, Kanto Chemical, Tokyo, Japan) in 200 μl of saline.

### Statistical analyses

Statistical evaluations of differences were performed with two-tailed unpaired Student's *t*-tests using SPSS statistics software (IBM Corp., version 22. Armonk NY, USA). Results with *p*-values of less than 0.05 were considered significant.

## Results

### Kinetics of infection with *P. yoelii* in GKO mice

**Figure 1:**
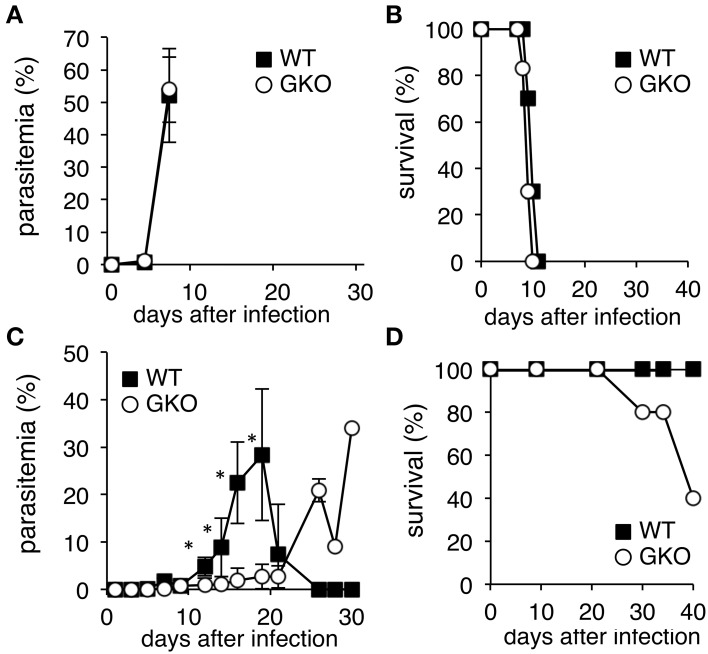
**Course of infection of** ***P. yoelii*** **in WT and GKO mice**. Parasitemias **(A,C)**, and survival rates **(B,D)** were monitored in WT (closed squares) and GKO mice (open circles) infected with either PyL **(A,B)** or PyNL **(C,D)**. Parasitemias were calculated based on microscopic observations of Giemsa-stained thin blood films. WT and GKO groups comprised five mice each. Values of parasitemia represent mean ± standard deviation. Asterisks denote statistical significance at *p* < 0.05 using Student's *t*-test. Similar results were obtained from three repeated experiments.

Given the hematopoietic roles of IFN-γ, we aimed to reevaluate the contributions of IFN-γ to the host-parasite relationship during malaria by infecting GKO mice with two strains of the rodent malaria parasite, *P. yoelii*. WT C57BL/6 mice infected with the lethal malaria strain, PyL, underwent a rapid elevation of parasitemia and died within 2 weeks (Figures 1A,B). Infection of GKO mice with PyL resulted in a similar course to that of WT mice, although the survival duration in GKO mice tended to be shorter (Figures 1A,B). In contrast, infection of WT mice with the non-lethal strain, PyNL, was non-lethal as expected, and the infection course showed a transient peak of parasitemia with complete resolution in 3 weeks (Figures 1C,D). Although all of the WT mice recovered from the PyNL infection, some of the infected GKO mice (16–60%) with high parasitemia died during the later phase of infection (Figures 1C,D), clearly indicating that the presence of IFN-γ conveys some protection. Unexpectedly, however, lower parasitemia was observed in the GKO mice than in the WT mice during the first 3 weeks post-infection (Figure 1C), suggesting that the presence of IFN-γ favors the growth of PyNL during early phase infection.

### Effects of IFN-γ on the production of reticulocytes during PyNL infection

We speculated that biological differences between the two tested malaria strains were the source of our observation that the GKO mice had lower parasitemia than the WT mice when they were infected with PyNL but not when they were infected with PyL. The major difference between these strains is their preference in erythroid host cells. PyL parasites infect a wide range of erythrocytes, but PyNL parasites preferentially infect younger cells, such as reticulocytes (Otsuki et al., 2009). Given our initial results and the difference in preferred host cell type of these two malaria strains, we hypothesized that IFN-γ affects the amount of host reticulocytes. To test this, we performed flow cytometric analyses using recombinant parasites expressing GFP, in combination with fluorescently-labeled antibodies to reticulocyte markers.

**Figure 2:**
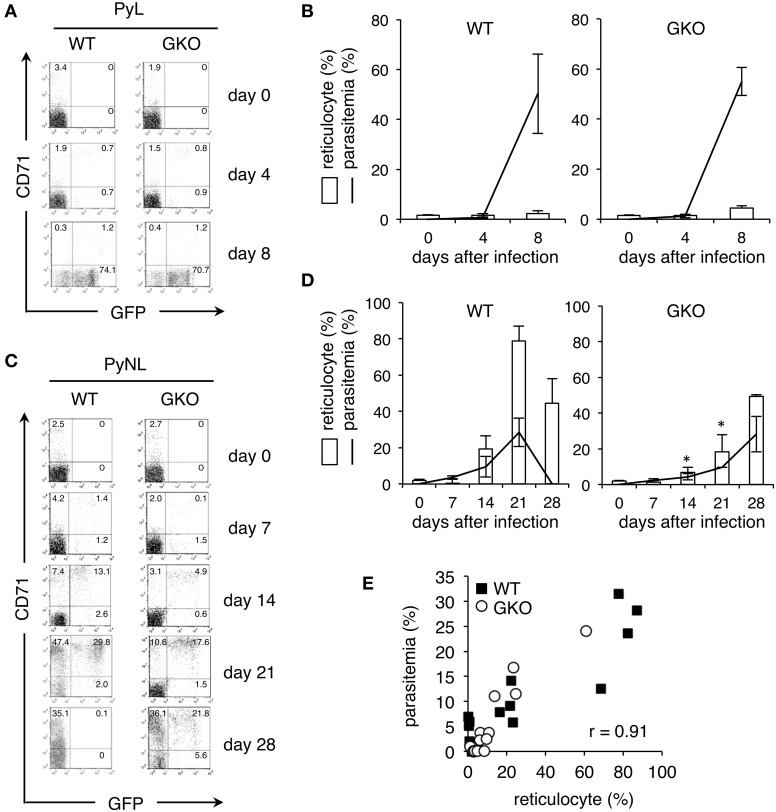
**Production of reticulocytes in WT and GKO mice infected with GFP-expressing** ***P. yoelii***. Flow cytometric analyses performed on peripheral blood from WT and GKO mice infected with GFP-expressing PyL **(A,B)** or PyNL **(C,D)**. Peripheral blood samples were stained with PE-Cy5.5-anti-TER119 and PE-CD71. One representative dot plot of gated TER119^+^ cells separated based on their expression of CD71 and GFP is shown for each condition. The numbers indicate percentages within each quadrant **(A,C)**. Bars or lines indicate percentages of CD71^+^ reticulocytes or GFP^+^ infected cells, respectively, among gated TER119^+^ cells at the indicated days after infection **(B,D)**. Values represent mean ± standard deviation from five mice. Asterisks denote statistical significance at *p* < 0.05 using Student's *t*-test compared with WT mice. **(E)** The percentages of parasitemia and the percentages of reticulocytes in peripheral blood from WT (closed squares) and GKO (open circles) mice infected with PyNL were plotted, and the correlation coefficient was calculated. The data used for this plot are from individual WT and GKO mice showing parasitemia during days 7–28 post-infection. Similar results were obtained from three repeats of the experiments.

Peripheral blood samples obtained from GKO mice infected with the recombinant parasites were stained with fluorescence-conjugated antibodies recognizing CD71, a transferrin receptor, and TER119, a marker for the late stages of murine erythroid lineage. We gated on TER119^+^ erythroid cells and considered those also expressing CD71 to be reticulocytes, as described previously (Koulnis et al., 2011). Prior to infection, GKO and WT mice had comparable amounts of reticulocytes (Figure 2). CD71^+^ reticulocytes were not increased in either the GKO or the WT mice during the entire course of infection with the recombinant PyL (Figure 2A), but as the parasitemia increased, GFP^+^ infected cells were detected in all of the infected mice. Both CD71^+^ and CD71^−^ cells were obviously infected at 4 days after infection, as indicated by their GFP expression, and CD71^−^ cells were overwhelmingly GFP^+^ at 8 days after infection (Figure 2A). No differences in parasitemia or reticulocyte numbers were observed between WT and GKO mice infected with PyL (Figure 2B).

In contrast to PyL infection, recombinant PyNL infection in WT mice gradually increased the amount of reticulocytes, whose increase was apparent at 14 days and reached its peak at 21 days after infection (Figures 2C,D). Interestingly, GKO mice infected with the recombinant PyNL parasites contained significantly fewer reticulocytes on days 0–14 post-infection than they did on day 21 post-infection. Once elevated, the level of reticulocytes remained high as late as 28 days after infection (Figures 2C,D). In both WT and GKO mice, the GFP^+^ infected cells were predominantly CD71^+^ reticulocytes, visually confirming the previously reported host cell preference. Parasitemia in WT and GKO mice infected with PyNL was closely correlated with their amount of reticulocytes (Figure 2E). These results suggest the that lower parasitemia observed in GKO mice infected with PyNL was due to these animals having fewer reticulocytes, and additionally, that IFN-γ is required for the induction of reticulocytes in the early phase of infection.

**Figure 3:**
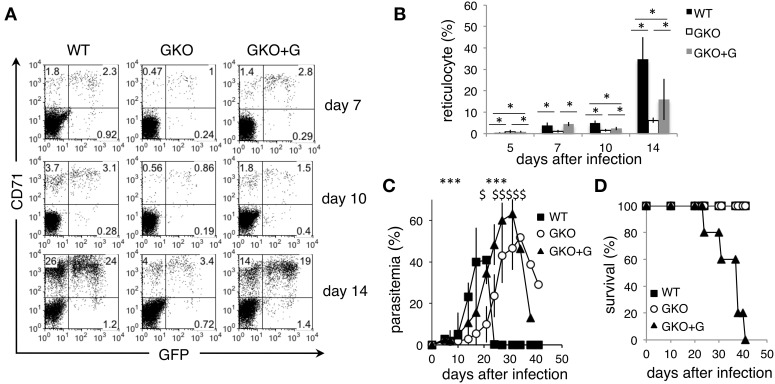
**Course of infection with PyNL in GKO mice injected with recombinant IFN-γ**. WT (closed squares) and GKO mice were infected with GFP-PyNL, and the GKO mice were left untreated (open squares) or treated with recombinant IFN-γ (GKO+G, closed triangles). **(A,B)** Blood samples were analyzed for reticulocytes infected with PyNL as in Figures 2C,D. Asterisks denote statistical significance at *p* < 0.05 using Student's *t*-test. Parasitemia **(C)** and survival rate **(D)** were monitored. Asterisks or sharps denote the statistical significance of the difference between values from GKO+G and those from control GKO or WT mice, respectively, at *p* < 0.05 using Student's *t*-test.

To further confirm the effects of IFN-γ on the course of PyNL infection, GKO mice were injected with recombinant IFN-γ. GKO mice that received injections of IFN-γ had significantly more reticulocytes both left uninfected and infected with PyNL than untreated GKO mice (Figures 3A,B). Furthermore, during the first 21 days post-infection, those mice had higher levels of parasitemia than untreated GKO mice (Figure 3C). Thus, these results clearly demonstrate that IFN-γ promotes parasite expansion by increasing reticulocytes during the early phase of PyNL infection. However, exogenous IFN-γ failed to confer resistance to the GKO mice, although WT survived the infection (Figure 3D). In this experiment, by contrast to the earlier results all GKO mice survived, suggesting that IFN-γ-independent protection may operate. These results demonstrate that the absence of IFN-γ during the late phase left these mice unable to control the higher parasitemia that resulted from the additional parasite growth in the presence of IFN-γ during the early phase, suggesting that IFN-γ-dependent responses are indispensable for resistance to a high parasite burden of PyNL during late phase infection.

### Kinetics of infection with PyNL in GKO mice treated with phenylhydrazine

**Figure 4:**
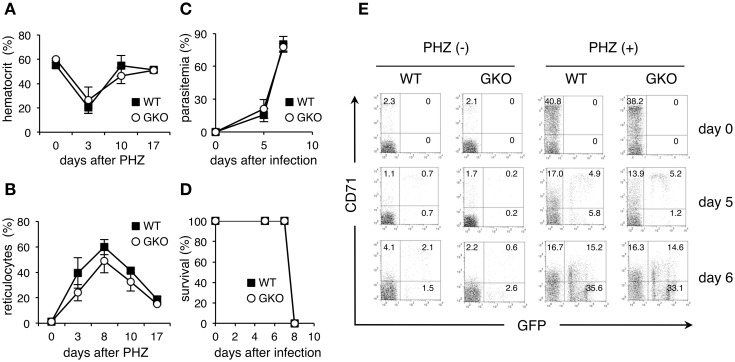
**Course of infection with PyNL in GKO mice treated with PHZ**. WT (closed squares) and GKO (open circles) mice were treated with PHZ and infected with GFP-expressing PyNL. Blood was taken from the tail vein at the indicated days after PHZ treatment and analyzed for the induction of anemia, as determined by hematocrit values **(A)** and for the amount of reticulocytes, based on flow cytometric analyses as in Figure 2B **(B)**. Mice were infected with PyNL 3 days after PHZ treatment, and parasitemia **(C)** and survival **(D)** were monitored as in Figure 1. Values represent mean ± standard deviation from five mice. Similar results were obtained from three repeated experiments. **(E)** Peripheral blood samples were stained with PE-Cy5.5-anti-TER119 and PE-CD71. One representative dot plot of gated TER119^+^ cells separated based on their expression of CD71 and GFP is shown for each condition. The numbers indicate percentages within each quadrant.

To further evaluate the possibility that the amount of reticulocytes present determines the course of PyNL infection, we infected mice that had been treated with PHZ, which induces hemolytic anemia. In both WT and GKO mice, injection with PHZ resulted in a transient reduction of hematocrit values and an increase in reticulocytes, presumably due to enhanced erythropoiesis in response to hemolytic anemia (Figures 4A,B). Infection of PHZ-treated WT mice with PyNL caused a rapid elevation of parasitemia and killed all the mice within 8 days (Figures 4C,D). GKO mice treated with PHZ also exhibited a sharp increase in parasitemia and succumbed to infection with PyNL similarly to the PHZ-treated WT mice (Figures 4C,D). Flow cytometric analyses confirmed that CD71^+^ GFP^+^ infected cells were noticeably increased both in WT and GKO mice as early as 5 days after infection. However, all these mice developed a large number of CD71^−^ infected cells as parasitemia increased (Figure 4E). Because enhanced erythropoiesis in PHZ-treated mice may increase not only CD71^+^ reticulocytes but also CD71^−^ younger erythrocytes as reported in individuals with iron-deficient anemia (Clark et al., 2014), it is postulated that in these PHZ-treated mice the PyNL parasites infected those younger cells rather than the mature erythrocytes. Indeed, infected CD71^−^ cells were observed even in untreated mice 7 days after infection with PyNL (Figure 4E). These results support the idea that PHZ treatment induces a reticulocytosis-mediated rapid expansion of PyNL parasites.

## Discussion

We demonstrated here that mice deficient in IFN-γ were more resistant to the early phase of infection with PyNL than WT mice, although they eventually developed higher parasitemia and some of them succumbed to an infection that is non-lethal in WT mice. Thus, we found that IFN-γ contributes to protection against infection with PyNL, which supports findings from previous studies. IFN-γ plays a protective role in malaria infection by inducing the activation of macrophages, which are responsible for the clearance of merozoites and parasitic erythrocytes (Yoneto et al., 2001; Su et al., 2002). It also drives humoral immunity by enhancing IgG2a production, as well as supporting cellular immunity (Waki et al., 1995). Our findings additionally indicate that IFN-γ-independent protective mechanisms successfully control mild parasitemia with PyNL observed in GKO mice, as some or even all GKO mice survived the infection (Figures 1A, 3A). However, the results of exogenous IFN-γ injection into GKO mice highlighted the importance of this cytokine; although WT mice cleared the high parasitemia, GKO mice could not control the “artificial” high parasitemia induced by a transient IFN-γ injection. Unfortunately, unlike PyNL, the parasite growth of PyL was too rapid for us to evaluate the contribution of IFN-γ appropriately in this infection model. However, we previously demonstrated the importance of IFN-γ for protection from PyL in mice that had received a transfer of protective CD8^+^ T cells obtained from WT mice that had been live-vaccinated with PyNL followed by two boosts with PyL (Imai et al., 2010).

In contrast to the protective role of IFN-γ described above, the presence of IFN-γ appears favorable for expansion of PyNL parasites during the early phase of infection. One potential explanation for this is that the parasites use IFN-γ for their growth as a part of the reported hijacking of host molecules by malaria parasites. These parasites have been shown to use host calpain, a cysteine protease, to digest parasitophorous membrane and host cell membrane during egress from parasitized erythrocytes (Chandramohanadas et al., 2009). They also use host peroxiredoxin to attenuate oxidative stresses during erythrocytic growth (Koncarevic et al., 2009). However, it is unlikely that IFN-γ is involved in the parasite hijacking of host molecules because γRKO mice infected with PyNL, whose production of IFN-γ is intact, showed a similar phenotype to GKO mice infected with PyNL (Supplementary Material). Therefore, IFN-γ enhances parasite propagation by providing some signals to host cells through the IFN-γ receptor.

Our results indicate that the absence of IFN-γ results in an impaired increase in reticulocytes after infection with PyNL. This finding suggests that IFN-γ, with its receptor expressed on a wide range of hematopoietic cells, including erythroid progenitors (Belyaev et al., 2010; De Bruin et al., 2014), positively regulates erythropoiesis. However, this contrasts with previous reports demonstrating suppressive effects of IFN-γ on erythropoiesis (McDevitt et al., 2006; Libregts et al., 2011). Interestingly, GKO mice showed an increase in their levels of reticulocytes during the later phase of PyNL infection. Additionally, reticulocytosis still occurred when GKO mice were treated with PHZ, indicating that IFN-γ-independent erythropoiesis may operate in such situations. Thus, IFN-γ-dependent erythropoiesis seems to be specific for the early phase of infection with PyNL.

As a result of the IFN-γ-mediated erythropoiesis in response to infection, the number of reticulocytes was increased in WT mice, allowing PyNL parasites to grow rapidly. The lack of IFN-γ in the GKO mice limited parasite growth, likely because of the associated reduced number of reticulocytes, given that these are the preferred host cells for PyNL parasites. Indeed, supplementation with exogenous IFN-γ to GKO mice raised parasitemia presumably due to an enhanced production of reticulocytes during the early phase of infection. Importantly, the parasite growth of PyNL closely correlated with the amount of reticulocytes throughout our experiments, including the high parasitemia we observed during hemolysis-induced reticulocytosis in mice treated with PHZ. Previous studies found that mice treated with recombinant IFN-α showed a reduction in the amount of reticulocytes and an inhibition of parasite growth of both *P. yoelii* 265BY and PyNL, both of which prefer reticulocytes as host cells, but not of *P. vinckei petteri*, which targets mature erythrocyte cells (Vigario et al., 2001). Thus, variation in the number of host cells may determine the course of infection. In these experiments, IFN-γ appears to support parasite growth by increasing the amount of reticulocytes. In another report, a supportive role for IFN-γ in the parasite growth of *P. yoelii* 265BY (Soulard et al., 2009) was attributed to erythropoiesis, although its effects on reticulocytes were not evaluated.

This study does not address how IFN-γ enhances erythropoiesis. As with all hematopoietic cells, erythrocytes are derived from hematopoietic stem cells that underwent stepwise differentiations into common myeloid progenitors, then into megakaryocyte-erythrocyte progenitors, and then into erythroid progenitors, like BFU-E and CFU-E. Finally erythroblasts emerged and, after enucleation in the bone marrow, became reticulocytes. These reticulocytes entered the circulation and shed residual RNA, developing into matured erythrocytes within 2 days. As reticulocytes are end products of the erythroid lineage, a disturbance in any of the above steps may affect the reticulocyte count. We found that the serum erythropoietin concentration was increased in response to infection with PyNL in GKO mice similarly to that in WT mice (data not shown). This hematopoietic factor acts specifically on erythroid progenitors, suggesting that the disturbance in GKO mice might exist upstream of BFU-E and CFU-E. During infection with PyNL, IFN-γ may activate hematopoietic stem cells as it was reported to do during bacterial infections (Baldridge et al., 2010); therefore, a lack of IFN-γ would reduce the number of hematopoietic stem cells. However, the numbers of leukocytes and thrombocytes were not altered in GKO mice infected with PyNL (data not shown), suggesting that the erythroid lineage (e.g., differentiation of megakaryocyte–erythroid progenitors to erythroid progenitors), rather than the multipotent stem cells, may be specifically affected in these mutant mice. Another possibility is that IFN-γ induces erythroblasts to express a (pro) renin receptor, a component of the renin–angiotensin system that enhances erythropoiesis (Vlahakos et al., 2010; Kaneko et al., 2012). Furthermore, given the ability of IFN-γ to activate macrophages, IFN-γ may promote the supply of erythroblasts by affecting erythroblastic islands where central macrophages play a crucial role (De Back et al., 2014).

IFN-γ production has been observed in several types of cells, including CD4^+^ Th1, CD8^+^ T, NK, NKT, and γ δ T cells (Inoue et al., 2013). Our preliminary experiments revealed that RAG2-deficient mice, which lack T and B cells, showed resistance during the early phase of PyNL infection similarly to GKO mice (data not shown). These results suggest that during PyNL infection, T cells are predominantly responsible for the production of IFN-γ.

In summary, we found that the role of INF-γ in hematopoiesis determines the course of infection with PyNL malaria parasites by affecting their preferred erythrocytic host cells, which could be deleterious for the host in terms of controlling parasite growth. Our findings provide novel insights into the interplay between host immunity and regulating erythropoiesis, and these results may help in understanding how IFN-γ functions in the host-parasite interaction during malaria infection.

## Author contributions

OH performed experiments, analyzed data, and wrote the manuscript. KS, TI, TT, CS, RO, and JH also performed experiments and provided advice. HH conceived the experimental ideas tested in these experiments and wrote the manuscript.

### Conflict of interest statement

The authors declare that the research was conducted in the absence of any commercial or financial relationships that could be construed as a potential conflict of interest.

## Abbreviations

PyNL: *P*.yoelii 17XNL
PyL: *P. yoelii* 17XL
GKO: IFN-γ knock out mice
γRKO: IFN-γ receptor knock out mice
PHZ: phenylhydrazine.
